# Obesity and Leptin Resistance in the Regulation of the Type I Interferon Early Response and the Increased Risk for Severe COVID-19

**DOI:** 10.3390/nu14071388

**Published:** 2022-03-26

**Authors:** Frits A. J. Muskiet, Pedro Carrera-Bastos, Leo Pruimboom, Alejandro Lucia, David Furman

**Affiliations:** 1 Department of Laboratory Medicine, University Medical Center Groningen, University of Groningen, 9713 GZ Groningen, The Netherlands; 2 Center for Primary Health Care Research, Lund University, 205 02 Malmö, Sweden; pmcbastos@gmail.com; 3 Faculty of Biomedical and Health Sciences, Universidad Europea de Madrid, 28670 Madrid, Spain; 4 Centro de Estudios Avanzados en Nutrición (CEAN), 11007 Cádiz, Spain; 5 PNI Europe, 2518 JP The Hague, The Netherlands; info@cpnieurope.com; 6 Pontificia University of Salamanca, 37002 Salamanca, Spain; 7 Faculty of Sport Sciences, Universidad Europea de Madrid, 28670 Madrid, Spain; alejandro.lucia@universidadeuropea.es; 8 Physical Activity and Health Research Group (‘PaHerg’), Research Institute of the Hospital 12 de Octubre (‘imas12’), 28041 Madrid, Spain; 9 Biomedical Research Networking Center on Frailty and Healthy Aging (CIBERFES), 28029 Madrid, Spain; 10 Stanford 1000 Immunomes Project, Stanford University School of Medicine, Stanford, CA 94305, USA; 11 Buck Artificial Intelligence Platform, The Buck Institute for Research on Aging, Novato, CA 94945, USA; 12 Edifice Health Inc., San Mateo, CA 94401, USA; 13 Austral Institute for Applied Artificial Intelligence, Institute for Research in Translational Medicine (IIMT), Universidad Austral, National Scientific and Technical Research Council (CONICET), Buenos Aires 2290, Argentina

**Keywords:** leptin, obesity, metaflammation, interferon, SOCS, COVID-19, SARS-CoV-2

## Abstract

Obesity, and obesity-associated conditions such as hypertension, chronic kidney disease, type 2 diabetes, and cardiovascular disease, are important risk factors for severe Coronavirus disease-2019 (COVID-19). The common denominator is metaflammation, a portmanteau of metabolism and inflammation, which is characterized by chronically elevated levels of leptin and pro-inflammatory cytokines. These induce the “Suppressor Of Cytokine Signaling 1 and 3” (SOCS1/3), which deactivates the leptin receptor and also other SOCS1/3 sensitive cytokine receptors in immune cells, impairing the type I and III interferon early responses. By also upregulating SOCS1/3, Severe Acute Respiratory Syndrome Coronavirus (SARS-CoV)-2 adds a significant boost to this. The ensuing consequence is a delayed but over-reactive immune response, characterized by high-grade inflammation (e.g., cytokine storm), endothelial damage, and hypercoagulation, thus leading to severe COVID-19. Superimposing an acute disturbance, such as a SARS-CoV-2 infection, on metaflammation severely tests resilience. In the long run, metaflammation causes the “typical western” conditions associated with metabolic syndrome. Severe COVID-19 and other serious infectious diseases can be added to the list of its short-term consequences. Therefore, preventive measures should include not only vaccination and the well-established actions intended to avoid infection, but also dietary and lifestyle interventions aimed at improving body composition and preventing or reversing metaflammation.

## 1. Introduction

The course of Coronavirus Disease-2019 (COVID-19) is very heterogeneous, ranging from asymptomatic [1] or mild through to severe disease and death [2]. Age, male sex, overweight/obesity, hypertension and type 2 diabetes are among the most important risk factors for severe COVID-19 [2,3,4,5]. Intriguingly, the highest rates of COVID-19 deaths are seen in high-income countries, which have the highest life expectancy and gross national product, but also a high prevalence of chronic diseases associated with an inactive lifestyle and obesity [6,7]. Interestingly, age and obesity also appear to be risk factors for post-acute COVID-19 syndrome (popularly labeled long COVID) [8,9,10]. This consists of the presence of at least one symptom, sign, or altered laboratory parameter beyond two weeks following acute infection, and is estimated to affect 80% of the infected patients with Severe Acute Respiratory Syndrome Coronavirus (SARS-CoV)-2, being more frequent in women than men [11].

From the onset of the pandemic, it was clear that obesity was a risk factor for severe COVID-19, which is not surprising, given that it was also a risk factor in the 2009 N1H1 influenza (“Mexican swine flu”) pandemic [12]. For example, of a sample of adult California residents who died of 2009 H1N1 infection, 61% suffered from obesity [13]. And in the SARS-CoV-2 predecessors, SARS-CoV-1 and Middle East Respiratory Syndrome *Coronavirus* (MERS-CoV), obesity and its associated comorbidities were also risk factors for a severe course [4,14]. Obesity is a known risk factor for many conditions, including type 2 diabetes, cardiovascular disease, cancer and various other diseases related to a poor lifestyle [15,16]. It is less well known that obesity also adversely affects the susceptibility to and outcome of infectious diseases [6,12,13,14,17,18,19,20,21].

This review attempts to find an answer to the question of why SARS-CoV-2 infection in people with obesity can lead to serious illness and death. Insight into the mechanisms, and thus the different phases, of the disease is of great importance for prevention and treatment, as is identifying the main risk factors. In that regard, it does not make much sense to make a sharp distinction between a number of risk factors, because they are strongly linked through chronic low-grade inflammation [22,23], also called “inflammaging”—when associated with aging [24]—and “metaflammation”—when it occurs in the context of the metabolic syndrome [25]. The latter, also called “insulin resistance syndrome”, which represents the common denominator of various COVID-19 risk factors [25,26,27,28], is a combination of excessive body fat, high blood pressure and impaired glucose and lipid metabolism [26,29].

As will be discussed in more detail, chronic low-grade inflammation, as well as elevated levels of leptin—a frequent observation in obesity—disrupts the immune system in a number of ways, increasing the risk for severe COVID-19, as well as for a severe course of other infectious diseases. Thus, when considering two patients having to deal with the same acute infection, the one with a pre-infection chronic state of metaflammation will endure a tougher challenge with increased probability of complications [19,20,22,23,30,31]. Noticeably, the mechanisms discussed in this review do not come solely from the study of SARS-CoV-2. Much was already known from research into other infectious microorganisms, in particular influenza viruses, but also SARS-CoV-1 and MERS-CoV. Obviously, the use of this data does not imply that all viruses are created equal [32,33], but aims at finding unity in pathophysiology.

## 2. Risk Factors for Severe COVID-19

Many risk factors for severe COVID-19 have been identified. In addition to viral load, age and male sex, these include obesity and its associated conditions, such as type 2 diabetes, cardiovascular disease, hypertension and chronic kidney disease [3,5]. Additional risk factors include genotype, socio-economic status, ethnicity, biological age, pregnancy, cancer/chemotherapy, allergy, asthma, chronic obstructive pulmonary disease (COPD), immune deficiencies, nutritional status, and the quality of the health care system [5]. Many of these risk factors are linked. Indeed, both obesity and age are positively associated with type 2 diabetes [16,34], insulin resistance [35,36], cardiovascular disease [16,37], and cancer [16,38]. Furthermore, the prevalence of micronutrient deficiencies, which is another potential risk factor for COVID-19 complications [39,40,41,42,43,44,45,46,47,48,49], is high among individuals with obesity [50,51,52,53,54], older adults [55,56,57,58,59,60], and also in severe COVID-19 patients [61,62,63]. Of relevance, lymphopenia, which is a known negative prognostic factor in severe COVID-19 [5], is also a marker of malnutrition [61]. In that regard, it should be mentioned that various studies from different countries, as reviewed by Bauer and Morley [64], have reported a significant prevalence of malnutrition in elderly patients admitted for COVID-19. Nevertheless, the influence of micronutrient deficiencies in the aging immune system (immunosenescence) and how it affects infection susceptibility and vaccination effectiveness in the elderly has so far been given little attention [65].

The link between severe COVID-19 and obesity, type 2 diabetes and metabolic syndrome can be illustrated as follows. In China, at the start of the pandemic, 14.7% of COVID-19 patients with a body mass index (BMI) < 25 kg/m^2^ from three hospitals had a severe and critical form, compared to 33.3% of patients with a BMI ≥ 25 kg/m^2^ (considered obesity in an Asian population) [66]. An increase in BMI of 1 kg/m^2^ corresponded to a 12% increase in the risk of a serious course. Obesity increased the risk of severe COVID-19 by about a factor of three and lengthened hospitalization times accordingly [66]. In France, relative to the general population, patients with severe COVID-19 in Lyon had a 1.35 times higher prevalence of obesity. And in patients with critical COVID-19, the prevalence of obesity was 1.89 and 2.88 times higher, in Lyon and Lille, respectively, in comparison with the French population [67]. Similar findings were found in a very large Spanish study (433,995 SARS-CoV-2 infected subjects; 25–79 years), where patients with severe obesity (BMI ≥ 40 kg/m^2^) were approximately 2.2 times more likely to be hospitalized and 2.3 times more likely to have severe COVID-19 [68]. Noticeably, in patients under 50 years of age, the chances of being hospitalized and suffering a serious course were even higher: 5 and 13.8 times greater, respectively [68]. This suggests that BMI is a stronger risk factor in young individuals. Accordingly, in American COVID-19 patients admitted to an intensive care unit (ICU), an inverse correlation was found between age and BMI [69]. Therefore, in a population with a high obesity prevalence, young individuals are more severely affected than is generally thought [69]. And there are now several systematic reviews and meta-analysis concluding that overweight and obesity is an important risk factor for COVID-19 severity [21,70,71,72,73], especially when it is characterized by excessive visceral adipose tissue [73,74,75,76].

Regarding obesity-related conditions, particularly type 2 diabetes, a meta-analysis indicated that it increases the risk of ICU admission by approximately 2.79-fold and mortality by a factor of 3.21 [77]. People with type 2 diabetes and metabolic syndrome also seem to be more susceptible to becoming infected. In a population-based U.S. study of 61.4 million adults between December 2019 and May 2020, 8885 (0.01%) had documented COVID-19 [78]. Individuals with metabolic syndrome had a much higher cumulative COVID-19 incidence than their counterparts without it (OR 7.0). For the individual components of metabolic syndrome, the adjusted odds ratios (aOR) for COVID-19 were: 2.5 (hypertension), 2.2 (obesity), 1.7 (hyperlipidemia) and 1.4 (diabetes). The incidence of COVID-19 was also higher in those with nonalcoholic steatohepatitis (aOR 4.93)—which is the hepatic manifestation of metabolic syndrome —and among African Americans (OR 7.45) [78], who have a high age-adjusted prevalence of obesity [79]. Similarly, and as it will be further discussed, individuals with obesity are more susceptible to various viral infections. Moreover, patients with both obesity and severe COVID-19 carry greater amounts of virus and also shed it for longer [12,80,81,82]. Furthermore, individuals with obesity show a muted immune response and are more likely to develop secondary infections, such as those caused by bacteria [17]. In addition, since the virus mutates faster with increasing virulence, it has been suggested, based on animal data regarding the influenza virus, that obesity may foster the emergence of new variants [82]. Finally, obesity is associated with the impaired effectiveness of vaccination [83,84,85,86] and antiviral drugs [12].

There are several explanations for the poor COVID-19 outcomes seen in obesity, including altered pulmonary physiology, predisposition to thrombotic complications, as well as difficulties in treatment in the ICU [2,6,20,41,87]. However, it is becoming increasingly clear that an altered immune system plays a major role [87]. One of the most important discoveries of recent decades is that inflammation and metabolism are intimately linked [25]. Inflammation (e.g., due to a viral infection) changes metabolism. Conversely, disturbances in metabolism (for example, due to obesity) can cause inflammation. Thus, it could be argued that a strict distinction between hormones and cytokines should be avoided. Moreover, no hormone or cytokine has just a single function and their functions overlap widely. Leptin is a good example of this [88,89], as will be illustrated below. This has led to the amalgamation of metabolism and inflammation into the term “metaflammation” [25], which is a hallmark of advanced age, obesity, type 2 diabetes and other risk factors for severe COVID-19 [4]. Metaflammation creates the perfect environment for an over-reacting immune system in the form of a “cytokine storm” [4], as is discussed further below. In essence, it is about what Darwin meant by “adaptations to the conditions of existence”. In order to ensure survival, inflammation causes a state of allostasis. Allostasis describes the adaptive process that, in response to predictable (e.g., pregnancy) or unpredictable (e.g., traffic trauma) disturbances, causes stability through change [90,91,92,93]. Resilience describes the ability to successfully respond to a challenge and return to a state of optimal adaptation (homeostasis). In line with the current definition of health [94,95], individuals with metaflammation have a reduced ability to “adapt and self-manage in the face of social, physical and emotional challenges” [94]. This is especially manifested when an organism is put under pressure by a major acute challenge, such as a microbial infection (e.g., SARS-CoV-2).

## 3. Cell Entry by SARS-CoV-2

SARS-CoV-2, like its predecessor SARS-CoV-1, enters the cell via the angiotensin-converting enzyme 2 (ACE2) [96,97,98,99,100,101]. This widespread enzyme is on the plasma membrane of many cell types [101,102], and acts as a receptor for the virus “spike” protein (S). The spike protein attaches to ACE2 through its receptor binding domain. The complex is then proteolytically processed by the transmembrane protease serine 2 (TMPRSS2) on the host cell, cleaving ACE2 and activating the spike protein [103]. It is possible that the fragments of the spike protein released during this process act as distractors of neutralizing antibodies directed against the spike protein [104,105]. Cells that can be infected carry both ACE2 and TMPRSS2 (or alternative proteases) on their membrane [103]. Their expression in various lung cells increases with age and is higher in males and smokers [102]. The SARS-CoV-2/ACE2 complex is then taken up via endocytosis [97,99,100,103]. This is a complicated process in which other proteases (e.g., furin) may also play a role [103]. In some cells, SARS-CoV-2 is first captured by the membrane protein neuropilin-1 and then forwarded to ACE2 [106].

ACE2 is encoded on the X chromosome [107]. Estrogen-induced or constitutional ACE2 expression takes place in females, but the resulting higher probability of SARS-CoV-2 entry into the cell is offset by higher androgen-driven expression of TMPRSS2 in males. The outcome of this interplay may explain the higher risk of severe COVID-19 observed in men [100]. Noticeably, individuals with obesity and type 2 diabetes appear to express more ACE2 [108]. And there is data showing that the visceral and subcutaneous adipose tissue actually has a higher ACE2 gene expression than the lungs [109].

Although ACE2 is found at higher levels on nose and bronchi epithelial cells and on pneumocytes [2,107], it is also expressed by endothelial cells [110], which helps to understand the effects of SARS-CoV-2 outside of the lungs [101]. Accordingly, a wide range of organs may become affected in COVID-19 [111], particularly the heart [112] and the brain [113,114,115,116]. Interestingly, the immune system may also suffer direct damage by the virus. ACE2 is found on natural killer (NK) cells and T cells, and deficiency of these cells is a hallmark of severe COVID-19 [107]. Peripheral blood leukocytes have a low expression of ACE2, while tissue macrophages have a high one [117], which opens the possibility that the latter cells could function as a “Trojan horse” and thereby actually facilitating infection [118]. The presence of ACE2 on adipocytes has led to the suggestion that the adipose tissue of COVID-19 patients with obesity acts as a sizeable SARS-CoV-2 reservoir [119]. Nevertheless, the entry of SARS-CoV-2 into organs is usually inferred from post-mortem examination by PCR (Polymerase Chain Reaction), which can lead to erroneous conclusions, since this technique does not demonstrate viable virus. Other explanations for the extra-pulmonary effects of COVID-19 include systemic inflammatory response, local thrombosis, and virus entry into the endothelium and immune cells [101,120]. Accordingly, in a hamster model of SARS-CoV-2 infection, the inflammatory response generated by virus replication at the site of entry (the upper respiratory tract) was found to trigger an inflammatory reaction that also affected distant organs, whether these contained viral particles or not [121].

## 4. Immune Defense Dynamics and Clinical Course

The antibody response to SARS-CoV-2 has a large inter-individual variation. IgM antibodies rise from approximately day seven after infection (seroconversion) and fall from day 16. As for IgG antibodies, these rise from day 14 and last much longer. Taken together, neutralizing antibodies reach their peak about 30–40 days after the onset of symptoms. Nevertheless, patients with severe COVID-19 achieve higher peak antibody titers and do so later (90 days) [122,123,124]. It has been found that the high titers of the anti-spike protein IgG in severe COVID-19 patients are associated with an aberrant glycosylation pattern of the Fc receptor [125]. The carbohydrate chains contain less of the monosaccharide fucose and more galactose. This composition renders the IgG more proinflammatory due to an enhanced induction of interleukin-6 (IL-6) and tumor necrosis factor-α (TNF-α) upon attachment of this IgG to (alveolar) macrophages. It explains the peak in inflammation, edema and thrombosis, and thus why patients get so sick, around the time of seroconversion [125]. The attached carbohydrate chains in glycoproteins are genetically and epigenetically determined and modulate the biological function of these proteins and the fate of the whole cell. The IgG carbohydrate chain is a known factor in the intensity of the proinflammatory response evoked by IgG. Probably not coincidentally, glycoproteins, including IgG, with aberrant carbohydrate chains have been linked to obesity, insulin resistance, obesity-induced hypertension, type 2 diabetes and other conditions associated with metabolic syndrome [126].

On average, admission to hospital occurs about 12 days after infection (about seven days after the onset of symptoms). It is important to realize that the virus is then (almost) no longer active. From that moment on, the most severe forms of disease are mostly driven by the immune system that has already caused extensive damage and keeps overreacting to virus residues (so called Pathogen-Associated Molecular Patterns or PAMPs) and cell debris (labeled Danger-Associated-Molecular-Patterns or DAMPs). This creates a “feedforward loop”, maintained by cytokines, which can lead to the infamous “cytokine storm”. This is characterized by fulminant inflammation, endothelial damage, thrombotic complications, life-threatening acute respiratory distress syndrome (ARDS), pneumonia, secondary infections, sepsis and multi-organ failure [4,110,111,127,128,129,130,131]. Before moving towards the immunology of severe COVID-19, the main features of the immune response are recalled.

## 5. Immune Response to a Pathogen in Short

The immune system orchestrates numerous responses that coordinately lead to the removal of a pathogen and the emergence of protection against any future encounters. If the various operational cascades are not perfectly attuned in terms of time and intensity, imbalances in the immune response may occur. This can cause major damage and is mechanistically an important, if not the main, cause of death in primary respiratory infectious diseases such as COVID-19 [20,127,132].

Briefly, the following takes place in an immune reaction. An initial and rapid response (hours) comes from the innate, non-specific, immune system. Sensors—also called “pattern recognition receptors” (PRRs), such as toll-like receptors (TLR)—on innate immune cells (such as dendritic cells and macrophages), as well as fibroblasts and epithelial cells, detect PAMPs. It may also be that released endogenous substances from damaged cells (DAMPS), such as ATP, are the first to be noticed by immune cells [127,133]. Recognition of PAMPs and/or DAMPs triggers a signaling cascade in which transcription factors, such as the NFκB (nuclear factor binding near the k light-chain gene in B cells), are activated, thus leading to an upregulation of inflammation-related genes. Pro-inflammatory cytokines and chemokines are then formed and secreted as carriers of messages to other cells. This acute inflammatory response can be amplified by the activation of inflammasomes (intracellular protein complexes) by DAMPs, which converts pro-cytokines, such as pro-IL-18 and pro-IL-1β, into biologically active cytokines—e.g., IL-18 and IL-1β, respectively. The secreted cytokines warn nearby cells to defend themselves by inhibiting virus replication, and to go into apoptosis (programmed cell death) if the cell is infected, and in their turn to secrete other inflammatory as well as anti-inflammatory cytokines. Recognized microorganisms are phagocytosed (by dendritic cells, blood monocytes, tissue macrophages and neutrophils) and protein fragments of the microorganisms are presented by dendritic cells to naive CD4+ T cells. These differentiate into T helper (Th) 1 cells (which stimulate cytotoxic T cells for cell-mediated immunity), Th2 cells (that stimulate B cells for humoral immunity), and other “effector” cells, especially Th17 and T-regulatory (Treg) cells, with pro and anti-inflammatory effects, respectively [134]. B cells become plasma cells (“effector B cells”) and these produce antibodies that can neutralize the pathogen. In this way, the adaptive immune system also provides longer-term protection starting within one to two weeks after infection in the form of neutralizing antibodies (humoral immunity), while cell-mediated immunity arises via cytotoxic CD8+ T cells that can kill pathogens [20,32,127,132].

In addition to the memory for making antibodies in the form of “memory B cells” [134,135], in the last phase of the infection, “memory T cells” [134,135] are also formed to be able to react more quickly in the event of a re-contamination [136]. Vaccination aims to build both forms of humoral and cellular immunity [137]. In SARS-CoV-2 infection, the formation of antibodies and memory B cells, CD4+ T cells, and CD8+ T cells each have their own dynamics [123,135]. Less well known is that the innate immune system can also provide a “cellular memory” which is called “trained immunity”. This memory is established as epigenetic marks and is useful in the very early phase of an infection [133,138,139].

Knowledge of the activation of the immune system, particularly T cell activation, is important for understanding what happens in a SARS-CoV-2 infection and how obesity and its related conditions could affect its course. Essentially, this involves a transition from a “dormant” (naive) state to rapid cell growth, multiplication and differentiation, aimed at promoting or inhibiting the immune response in the coordinated manner described above. These so-called effector cells consist of T helper cells (Th1, Th2 and T17; which arrange the immune response) and regulatory T cells (Treg; which inhibit the immune response). Crucial in the activation of the “dormant” (naive) T cells and also of pro-inflammatory monocytes is the acquisition of energy—immunometabolism. The first step is the uptake of glucose. For this purpose, the immune system contains several of the 14 currently known glucose transporters (GLUT), GLUT1 being the most relevant [140]. This must be expressed for the ultimate functionality of the pro-inflammatory T effector cells, such as Th1, Th2 and Th17 [140,141]. Accordingly, GLUT1 deficient T effector cells cause an ineffective inflammatory response and GLUT1 overexpression causes inflammatory disease [140]. Interestingly, the immune-suppressing Treg cells are not dependent on GLUT1 [140]. Inadequate expression of GLUT1 in the immune system is probably the most important link between SARS-CoV-2 infection and the development of severe COVID-19, as will be explained later.

## 6. SARS-CoV-2 Disrupts the Early Immune Response: Interferons

**Figure 1:**
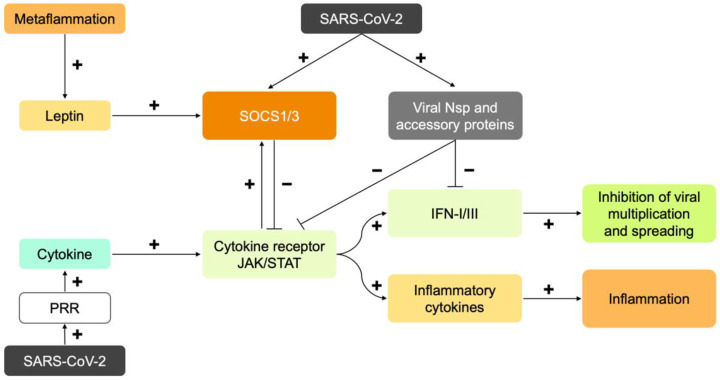
Schematic representation of the immune response and its dysregulation by metaflammation and SARS-CoV-2. The virus is recognized by pattern recognition receptors (PRR). PRRs are located on the membrane and in the cytoplasm. Through the secretion of cytokines, the infected cell warns other cells to inhibit virus multiplication and spreading (via interferons; IFN-I and -III) and initiate an inflammatory response (via inflammatory cytokines such as TNF-α and IL-6). Signal transduction by cytokines takes place via a cytokine receptor and the Janus kinase/Signal Transducer and Activator of Transcription protein (JAK/STAT) pathway. This pathway creates its own boundary by also expressing the proteins “Suppressor of Cytokine Signaling 1 and 3” (SOCS1/3). These inhibit the JAK/STAT pathway via negative feedback. Metaflammation is characterized by chronic low-grade inflammation. In obesity, the inflamed adipose tissue chronically secretes pro-inflammatory cytokines, including leptin. Leptin and insulin resistance are generated through the inhibition of the JAK/STAT pathway by expression of SOCS1/3. Therefore, in metaflammation, there is already a brake on cytokine signaling before infection. The RNA of SARS-CoV-2 contains codes for non-structural (Nsp) proteins and “accessory” proteins. Infected cells express these proteins. They inhibit the induction and activation of IFN-I. The virus also causes the expression of SOCS1/3. The inhibition of IFN-I signaling by the virus, virus-mediated induction of SOCS1/3, and the preexisting elevation of SOCS1/3 in metaflammation, collectively cause late and initially weak IFN-I and -III responses, and a strong multiplication and spreading of the virus. Major damage is caused by the virus and the immune system. The resulting immune system overreaction, cytokine storm and hypercoagulation are hallmarks of a severe COVID-19 course.

The pathophysiology of COVID-19 involves many elements which are highly interrelated [5]. In short, these include the direct damage by the virus, the secondary damage by the immune response, the damage to the endothelium and the micro-vasculature, the hypercoagulability with in situ thrombosis and macrothrombosis, the already mentioned disruption of the ACE2 pathway, the dysregulation of the immune system, and the development of a hyperinflammatory state and a possible cytokine storm [9,127]. Viruses have developed strategies throughout evolution to disrupt the immune system and thus promote their multiplication and spreading. They thereby increase their virulence, which is the ability of a pathogen to cause damage. SARS-CoV-2 does so in at least two ways, as depicted in Figure 1.

In addition to the codes for the proteins in the spike (S), envelope (E), membrane (M) and nucleocapsid (N), the genetic material of SARS-CoV-2 (RNA) also contains the codes for 16 non- structural proteins (Nsp) and 9 “accessory” proteins, of which at least 10 interfere with both the induction and antiviral activity of interferons, especially interferon types I and III (IFN-I and III) [32,81,142,143,144,145]. The interferon system includes a series of antiviral cytokines, which are classified into types I, II and III based on their molecular structure and receptors [146,147,148]. Interaction with their respective receptors causes the expression of hundreds of “effector genes” (“interferon stimulated genes”; ISGs), which have antiviral and immuno-modelling functions [148]. Mechanistically, the antiviral properties of interferons are mediated by inhibiting several steps in virus replication. Inhibition of the interferon response by the virus is detectable in the bronchial cells and plasma of symptomatic COVID-19 patients, among others [142,144].

Normally, the cooperation between the myeloid (neutrophils, monocytes and macrophages) and lymphoid (including natural killer cells) lineages of the innate immune system leads to a rapid elimination of most of the microorganisms that infect humans every day. Only if that fails, due to a massive attack or a highly invasive pathogen, does the adaptive immune system (B and T cells) become significantly activated [149]. And that is exactly what SARS-CoV-2 strongly promotes. By disrupting interferon signaling, immediately after infection, the immune system loses its ability to prevent rapid multiplication and virus spreading. By the time it finally reacts, its only option is to mount a highly damaging immune response, which is also very energy-consuming. Thus, an infected host is initially dealing with a virus-induced impaired antiviral defense via the innate immune system, and a conserved inflammatory capacity driven by pro-inflammatory cytokines. The virus can multiply rapidly and trigger an enhanced inflammatory response via TNF-α, IL-6, and other inflammatory cytokines. In high risk groups (see below) this can lead to serious illness due to the development of the already mentioned “cytokine storm”. Disruption of the interferon response is not a unique strategy of SARS-CoV-2, but is also used, for example, by its predecessors SARS-CoV-1 and MERS-CoV [144]. However, SARS-CoV-2 is extremely effective in this regard [32].

The importance of interferons can be inferred from studies conducted in individuals with genetic defects in IFN-I synthesis or with autoantibodies to IFN-I [150,151]. If infected, they have a much greater chance of serious COVID-19 [132]. It has been estimated that up to 14% of patients with life-threatening COVID-19 have a genetic or autoimmune IFN-I deficiency [144]. In a study by Bastard et al. neutralizing autoantibodies to IFN-I were found in 10.2% of patients with life-threatening COVID-19 and in 0.33% of healthy controls. Intriguingly, these antibodies were already present before the infection [151]. In addition, SARS-CoV-2 infection is linked to the development of autoantibodies against components of the immune system and various other organs, and the development of autoimmune diseases [152,153]. Autoantibodies may be one of the explanations for the observed damage to a variety of organs, and it may represent another mechanism by which obesity increases COVID-19 severity risk. In accordance with this, a recent study with COVID-19 patients found that those who had obesity, when compared to lean ones, had higher levels of IgG autoantibodies against malondialdehyde (a biomarker of lipid peroxidation), and adipocyte-derived protein antigens (presumably due to virus-induced cell death in the adipose tissue). Moreover, the patients with obesity had significantly lower levels of anti-Spike IgG versus lean patients. Finally, few COVID-19 patients with obesity had neutralizing antibodies, while all of their lean counterparts had it [154].

There is a second way viruses may disrupt IFN-I signaling (Figure 1). This happens because they trigger the host to induce the proteins “Suppressor Of Cytokine Signaling 1 and 3” (SOCS1/3). Possible instigators are viral proteins, viral RNA, or the host’s own cytokine response [155]. Actually, this second way cannot be properly distinguished from the first, because the ultimate target, the activity of interferon signaling, is the same. Increased SOCS1/3 expression has been established for SARS-CoV-1 and MERS-CoV, but, to the best of our knowledge, not yet formally for SARS-CoV-2 [156]. SOCS1/3 are “hijacked” by many viruses to disrupt the immune response for their own purposes. Examples are the causative agents of influenza A, dengue, Zika virus disease, West Nile disease, Ebola virus disease, hepatitis B, AIDS and Pfeiffer’s disease (Epstein Barr Virus). They thereby increase their virulence [155,156,157,158,159,160,161,162]. Infectious gram positive and negative bacteria and mycobacteria also cause increased SOCS expression [163].

SOCS1/3 are “checkpoint” regulators of the immune system. They ensure that the immune system does not react too strongly to exogenous antigens or self-antigens (preventing autoimmune disease). The importance of this “internal control” can be seen when SOCS1/3 are turned off in a “knock-out” animal model. SOCS1 knockout causes neonatal death from an unregulated inflammation and SOCS3 knockout results in embryonic death [156]. One of the functions of SOCS1/3 is inhibiting leptin signal transduction, as will be discussed later. This results in immune suppression by Treg cells [157]. Ultimately, increased SOCS1/3 expression inhibits the production of interferon types I and II [164,165]. It turns out that SOCS1/3 are elevated in metaflammation [166], and that the interferon part of the immune response is therefore already suppressed prior to infection. As will be explained in more detail later, this is a major link between obesity and the development of severe COVID-19.

It is now clear that the disruption of the early innate immune response is an important strategy of SARS-CoV-2. However, that does not explain why most people escape severe COVID-19 and the risk groups apparently do not. To understand this heterogeneity, it is important to understand how the immune system is activated during an infection and why this response goes wrong and leads to the development of severe COVID-19. The bottom line is that what the virus does to disrupt the immune system was already disturbed in individuals with obesity before they got infected. In this scenario, COVID-19 represents a double dose of immune disruption.

## 7. Immune Response to SARS-CoV-2 in Obesity: Too Late, Too Weak, and Then Too Strong

**Figure 2:**
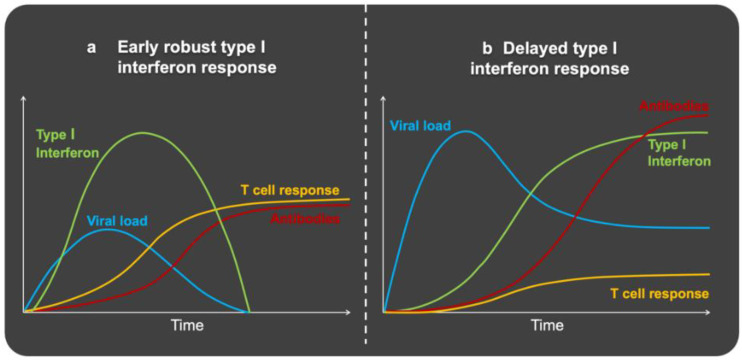
Schematic representation of immune response leading to (**a**) mild COVID-19 and (**b**) severe COVID-19. (**a**) Mild COVID-19 produces an early and robust type I interferon response. This limits the multiplication of the virus and its spreading. There is an adequate T-cell response and antibody formation. This type of response is characteristic of young people and also occurs when they are exposed to a low amount of virus. (**b**) In severe COVID-19, the type I interferon response is delayed. This gives the virus the chance to multiply and spread quickly. There is also an inadequate T cell response with low T cell numbers. Patients with severe COVID-19 achieve higher peak antibody titers and at a later time. This type of response is characteristic of the elderly and persons with metaflammation in general. It also occurs when they are exposed to a high viral load. Adapted by permission from Springer Nature: Nature Reviews Immunology (The First 12 Months of COVID-19: A Timeline of Immunological Insights, Carvalho, T.; Krammer, F.; Iwasaki, A., 2021) [132], available at https://www.nature.com/articles/s41577-021-00522-1 (License Number: 5254880681651, accessed on 23 February 2022).

Compared to low-risk groups, high-risk groups show a slower interferon response. This allows the virus to multiply and spread even faster, leading to “virus-induced pathology” [167], which triggers an exaggerated inflammatory response that causes sizeable collateral damage. Due to the cell debris (DAMPs) released during this process, an additional sterile inflammatory reaction arises. An already mentioned “feedforward loop” develops in which clotting is also excessively stimulated [168]. From an evolutionary perspective, coagulation can be seen as belonging to the innate immune system, since local coagulation immobilizes the pathogen [169]. At necropsy, platelet-rich thrombi are seen in the microvasculature of the lungs, liver, kidneys and heart of COVID-19 patients. In addition, the lungs show, among others, diffuse alveolar damage with hyaline membranes, hemorrhages and virus particles in epithelial cells and macrophages [170,171,172,173]. Figure 2 [132] shows the kinetics and intensity of the antiviral response after a SARS-CoV-2 challenge. With a mild to moderately severe COVID-19 course (Figure 2a), an early intense interferon type I response occurs, leading to a rapid decrease in virus load. This prevents lymphocyte counts from falling and pro-inflammatory cytokines from rising excessively. A serious course (Figure 2b) is characterized by a delayed interferon type I response, a high viral load, a weak lymphocyte response and a strong increase in pro-inflammatory cytokines and in antibodies [132,174].

How the immune system in the context of excessive adiposity responds to an infection has been previously described in animal studies. Seminal work from Smith et al. [175] showed that obese mice infected with a mouse-adapted influenza A virus exhibited increased mortality and were more susceptible to lung pathology than their lean counterparts. A central finding involved a downregulation of the type I interferon (e.g., IFN-α and IFN-β) response. At baseline, obese compared to lean mice had similar insulin levels, but elevated glucose and leptin concentrations, and they were insulin and leptin resistant. After challenge, major differences were observed at latter time points in the levels of insulin, glucose, and leptin, indicating that the regulation of endocrine factors and inflammatory events are largely dysregulated in obesity. Furthermore, mortality rates differed between the two groups, with a death rate of 42% in obese vs. 6% in lean animals on day eight after challenge, and this correlated with viral load in the lungs. Interestingly, induction of antiviral cytokines, such as IFN-α and IFN-β, was delayed and strongly reduced in the obese group. In contrast, lean mice exhibited a fulminant IFN-α and IFN-β response on day 3, which largely disappeared by day 6. Since interferons are responsible for the activation of NK cell cytotoxicity, this provides a mechanistic explanation for the lack of an effective response in metaflammation. Indeed, on day three post-challenge, NK activity was significantly reduced in the lungs and spleen of obese mice. This correlated with a reduced expression of pro-inflammatory cytokines, such as IL-18, IL-6, TNF-α and IL-1β. Importantly, in obese mice the inflammatory response only appeared in the late phase (day 6). Similar responses were seen for the chemokines MCP-1 (Monocyte chemoattractant Protein-1) and RANTES (Regulated upon Activation, Normal T Cell Expressed and Presumably Secreted), but not for MIP-1α (Macrophage inflammatory protein-1α), all of which are needed for the recruitment of immune cells to the site of infection. Therefore, in obesity, there appears to be a delay in the inflammatory response and in the recruitment of immune cells to the site of infection.

A similar experiment with diet-induced obesity in mice was conducted in 2013 by Zhang and co-workers [176]. Infection occurred with influenza A (H1N1), and the results were highly similar to results from Smith et al. [175]. For example, leptin levels of the obese mice were already strongly elevated before the infection. In this study, some of the obese mice were also treated with an anti-leptin serum, which resulted in a greatly improved survival (80 vs. 40%) and a reduction in the pro-inflammatory cytokines IL-6 and IL-1β in the lungs.

Taken together, these studies show that influenza A-infected obese mice exhibit a significantly higher mortality. They have reduced NK cell cytotoxicity and delayed IFN-α and IFN-β responses. This explains the insufficient inhibition of virus multiplication and spreading due to impaired interferons I and III responses. In the late phase of infection, the obese mice show a strong increase in pro-inflammatory cytokines, resembling a cytokine storm. The elevated leptin levels in the obese mice play a causal role in the severity of infection. The picture is very similar to what happens in humans with severe COVID-19. This requires a more in-depth discussion on the important role of insulin and leptin in the immune response.

## 8. Role of Insulin and Leptin in the Immune System: Immunometabolism

An inflammatory reaction consumes high amounts of energy [177]. Depending on its strength and duration, the additional energy costs of systemic inflammation are about 25–60% of the total energy expenditure of a healthy person [178,179,180]. In nature, energy is a scarce commodity. Therefore, there must be a good reason to initiate an inflammatory response. T cells have a critical role in immunometabolism [177]. As explained, one of the first steps here is the activation, multiplication and differentiation of naive T cells into “effector cells” [134,177,181]. This requires a high level of energy. Immune cells obtain most of their energy from glucose and glutamine [179]. As mentioned, the uptake of glucose in immune cells mainly occurs by GLUT1 in an insulin-independent manner [140]. Yet it turns out that T cells need an insulin receptor to become activated. This became clear when the insulin receptor of T cells was switched off in animal models, and selective T cell functions were affected. Glucose transport and glycolysis were reduced, and these metabolic disturbances were associated with decreased cytokine production, as well as impaired T cell proliferation, migration and response to antigens. These cells also showed increased apoptosis [182].

Consistent with these experiments, mice with insulin receptor-deficient T cells showed reduced inflammatory potential and poor protective immunity when infected with H1N1 influenza [183]. The behavior of T lymphocytes without an insulin receptor has a clear link with one of the strongest features of severe COVID-19, which is a high neutrophil/lymphocyte ratio, where the number of neutrophils is increased, and the number of lymphocytes is strongly reduced [184,185] (Figure 2b). This ratio is a surrogate for systemic inflammation, being associated with the metabolic syndrome [186]. It is a marker for low-grade inflammation, metaflammation and, in COVID-19, a marker for poor prognosis [187]. Compared to healthy controls, in patients with type 2 diabetes, glucose uptake into peripheral blood lymphocytes is slow and quickly reaches a plateau [188]. Insulin resistant individuals suffering from obesity have activated circulating neutrophils, monocytes and T lymphocytes [189,190]. Their T cells are of the pro-inflammatory type (Th1 and Th17) [183,190] and their in vitro exposure to insulin does not cause differentiation to an anti-inflammatory type (Th2 and Treg) [190,191]. Obesity therefore involves activated, insulin-resistant, immune cells.

Persistent in vitro and in vivo exposure of macrophages to insulin, which could occur in chronic hyperinsulinemia (a condition associated with obesity and the metabolic syndrome), renders them insulin resistant and produces an M2 phenotype that is less responsive to stimuli. M2 macrophages lower inflammation and promote tissue repair, but in the event of an acute infection they render the host more susceptible to invasion [192]. On the other hand, insufficient glucose metabolism in T cells is linked to what has been termed in immunology as “anergy” and (bioenergetic) “exhaustion” [30,87]. It therefore seems that in metaflammation, both macrophages and T cells are affected, with the latter not receiving sufficient energy (read: glucose) to be able to respond to an infection. Remarkably, this resembles what occurs in Alzheimer’s disease and other neurodegenerative diseases that are also linked to metaflammation and insulin resistance, where the affected parts of the brain do not gain enough glucose [193,194].

As mentioned, glucose is required for T-cell activation and function. Accordingly, naive T cells contain virtually no GLUT1. The absorbed glucose is not only required for energy, but also for the synthesis of phospholipids, proteins (including cytokines) and components of DNA and RNA, for the purpose of cell proliferation and differentiation, as well as the performance of specific effector functions [195,196]. Upon activation, major changes take place in immunometabolism. In order to be able to synthesize the aforementioned building blocks and also the antioxidant glutathione, the activated cells use glycolysis (glucose to lactate), which is also called the “Warburg effect” [197]. If glucose in T cells is mainly taken up via GLUT1, the question is why GLUT1 does not function. It turns out that leptin is responsible for GLUT1 expression.

Leptin is largely derived from adipose tissue. It provides the brain with information about the energy stores in the body, namely fat reserves. There is a direct relationship between the size of the adipose tissue compartment (measured as BMI) and circulating leptin and insulin levels [198,199]. BMI also correlates with inflammatory markers, such as acute phase proteins, TNF-α receptors and plasminogen activator inhibitor-1 (PAI-1) [198]. As a hormone, leptin induces satiety and increases energy expenditure. These characteristics are known as its effects in the CNS [200]. Less well known is that leptin is also a pro-inflammatory protein with an important role in the immune system [89,200,201]. Low leptin is related to malnutrition and an increased risk of infection, while high leptin correlates with obesity, leptin resistance, low-grade inflammation, neurodegenerative and autoimmune diseases [88,200], an M1 macrophage phenotype, decreased lymphocytes, and a severe course of COVID-19 [202,203].

By mediating GLUT1 expression, leptin grants activated T cells “permission” to take up glucose and initiate an energy-consuming immune response [177,195,204]. This “contributing” function of leptin is evident in malnourished or starving people, where low leptin (low reserves) is observed, which goes together with a high risk of infections [177,205]. The glucose uptake and glucose metabolism in their T cells is decreased, hence reducing T cell proliferation. These individuals also produce fewer cytokines and have a reduced capacity to respond to cytokines. They are characterized by an anti-inflammatory and immuno-tolerance state, and are therefore at a higher risk of infectious diseases and less likely to develop autoimmune diseases, respectively. Their low leptin prevents the initiation of an energy-demanding immune response. Accordingly, treatment of fasting animals with leptin restores glucose metabolism in their T cells [177].

Consistent with a crucial role of leptin signaling in the initiation of an immune response, laboratory animals and humans that do not make leptin due to a genetic defect have reduced T cell numbers, decreased production of pro-inflammatory cytokines and a decreased sensitivity to T cell activation. In these subjects this leads to an increased susceptibility to intracellular infections such as those caused by viruses. Similar defects are seen in mice with a genetic deficiency of the leptin receptor. In the case of genetic leptin deficiency, the immune defects can be reversed by treatment with recombinant leptin. Leptin is therefore an important regulator of a pro-inflammatory response. It forms the link between nutrition and immunity because it directly increases glucose metabolism in T cells and thus takes the first step towards the proliferation and differentiation, and hence the functionality, of effector T cells [177].

Given the above, it seems clear that insulin and leptin serve key functions in the link between diet and the immune system. Because of their disruption in obesity and metabolic syndrome, these two hormones are central to the development of severe COVID-19 in persons with metaflammation. By altering the metabolic setpoint, hyperleptinemia and insulin resistance disrupt T-cell function, leading to a suppressed T-cell response upon infection (see below). To understand the mechanisms, a brief explanation on how cytokines and leptin fulfill their task in the immune system is in order.

## 9. Cytokines as Immune System Messengers: JAK/STAT Signal Transduction

**Figure 3:**
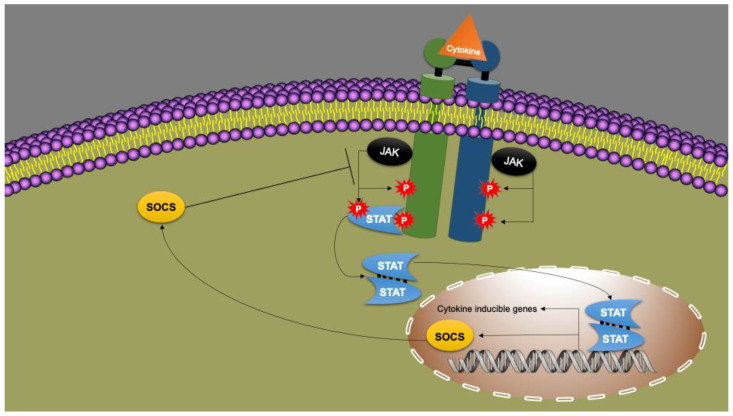
Cytokine signaling via the JAK/STAT pathway. A cytokine, such as leptin, binds to its receptor on the plasma membrane. The cytokine receptor is associated with the Janus kinase (JAK; “Just Another Kinase”). JAK phosphorylates two receptor tyrosines, after which JAK is recognized by STAT (Signal Transducer and Activator of Transcription). Subsequently, STAT is also phosphorylated by JAK. Two phosphorylated STATs form a dimer. The STAT dimer acts as a transcription factor. It binds to the promoter parts of genes affected by that cytokine (“target genes”). These genes are then transcribed into mRNA (DNA to mRNA) and translated into proteins (mRNA to protein). Proteins expressed via this JAK/STAT pathway have functions in proliferation, differentiation, growth and apoptosis. Together they form the products of the “Cytokine Inducible Genes” (CIG). Suppressor Of Cytokine Signaling (SOCS) is also a CIG product. SOCS inhibits the JAK/STAT pathway and thus cytokine signaling. This creates a negative feedback loop. Modified from Morris, Kershaw and Babon [147].

Leptin and various cytokines (such as IL-2, IL-6, and IFN-I, -II and -III) signal via the so-called JAK/STAT (Janus kinase/Signal Transducer and Activator of Transcription protein) pathway [147,205,206] (Figure 3).

With exceptions, each cytokine binds to a specific receptor, located on the plasma membrane of the target cell (Figure 3). The cytokine receptor is associated with Janus kinase (JAK). In response to the binding of a cytokine to its receptor, the JAK phosphorylates two tyrosines that are part of the receptor. JAK is now recognized by STAT, which is subsequently phosphorylated by JAK as well. Together with a second phosphorylated STAT, a dimer is formed. There are four different JAKs and seven different STATs. The resulting STAT dimer acts as a transcription factor, binding to the promoter region of the target genes of that cytokine, thereby leading to the synthesis of proteins that affect proliferation, differentiation, growth and apoptosis. Together, these proteins form the products of the “cytokine inducible genes” (CIG). Thus, in this way, the cytokine changes the phenotype of the target cell and the immune response becomes regulated and coordinated [147,206].

The JAK/STAT signal transduction is strictly controlled by SOCS proteins. As mentioned, the activation of the immune system implies an imminent consumption of large amounts of energy and can elicit significant damage to the host. Therefore, having mechanisms capable of slowing down or even stopping the immune response at the right time is crucial. This is the supposedly evolutionary basis for SOCS, which are proteins that act as “checkpoint” regulators. There are seven of these and another one called CIS (Cytokine-Inducible SH2 protein). SOCS-1/3 have been previously mentioned as a target of viruses. They are also crucial in obesity. SOCS is a product of the above-mentioned “cytokine inducible genes” (CIG). Thus, under the influence of inflammatory cytokines, including leptin, SOCS are expressed in the same way via JAK/STAT signaling. SOCS, however, disable the JAK/STAT signaling as the cytokine receptor is degraded by SOCS. In this way, SOCS3 switches off the leptin receptor and also other cytokine receptors that are sensitive to it. It is a negative feedback loop in which the cytokine signal via JAK/STAT limits itself and even switches itself off [147,206].

SOCS proteins, especially SOCS1/3, play a central role in the development of severe COVID-19. If they are induced for whatever reason (e.g., metaflammation) the normal immune response does not proceed.

## 10. Obesity and Severe COVID-19

Obesity and metabolic syndrome not only play a major role in the development of chronic diseases, but also have a strong influence on the immune system and the way it responds against pathogens. Various lines of evidence have shown that these conditions are characterized by a disturbance in the integrity of lymphoid tissue, changes in the development, phenotype and activity of leukocytes, and in the coordination of innate and adaptive responses. This manifests itself in a higher risk of infectious complications [207] and a lower vaccination responsiveness [83,84,85,86]. Accordingly, even after influenza vaccination, adults with obesity have a two-fold higher risk of influenza compared to healthy weight vaccinated counterparts [18]. Obesity is a risk factor for many infectious diseases, including COVID-19 [6,18,208,209,210]. The strong link between the COVID-19 pandemic and the pre-existing obesity pandemic has led to a contraction to “CoVesity” [211]. As mentioned, in obesity there is typically a basal state of metaflammation associated with insulin and leptin resistance which derails if an acute inflammatory reaction becomes superimposed by infection.

The influence of obesity on the immune system is considered to be a form of accelerated aging characterized by “thymus involution”, T-cell “aging” and T-cell “exhaustion” [27,212,213,214,215]. This view is based on observed differences between the immune system of the young and the elderly, having led to the often-cited term immunosenescence. This is poorly defined in terms of irreversible dysfunction, ill health and survival [216,217]. It fails to take into account disparities in basal health status and the modifiable factors that could affect those differences, leading to the question as to whether immunosenecescence is simply a consequence of the normal aging process or is in fact due to the life-long adoption of a faulty diet and lifestyle [218]. Giving support to the latter, Bencivenga and co-workers hypothesized that the correction of nutritional deficiencies may attenuate age-dependent changes in the innate and adaptive immune system [219]. Similarly, both obesity and type 2 diabetes can be reversed through lifestyle changes [220]. Nevertheless, this discussion is far from settled, and gaining insight into the underlying mechanisms that disrupt the immune system in obesity could help solve it.

### 10.1. Obesity, Metaflammation and Combined Leptin and Insulin Resistance

Adipose tissue is not only a storage and distribution center for energy. It is considered to be the largest endocrine organ and plays a crucial role in inflammation and the overall immune response. Obesity comes with a large adipose tissue compartment that may consist of 40–60% inflammatory macrophages [177,221]. Noticeably, the visceral adipose tissue (VAT) appears to be more pathogenic than subcutaneous depots, in part because its expansion occurs mainly through adipocyte hypertrophy, leading to local compression and hypoxia, which in turn promotes oxidative stress, cell apoptosis and necrosis. This, respectively, activates and recruits resident and non-resident immune cells, eliciting both a local and systemic low-grade inflammatory state [222,223,224] which is characterized by moderately elevated levels of C-reactive protein (CRP) and pro-inflammatory cytokines such as TNF-α and IL-6, as well as a slightly increased number of pro-inflammatory neutrophils and mast cells and a decreased number of anti-inflammatory natural killer T cells and eosinophils [177]. Therefore, it is not surprising that visceral adiposity has been associated with a higher risk of severe COVID-19, according to two systematic reviews and meta-analyses [74,225].

In the above scenario, the adipose tissue has become a significant source of inflammation, secreting increased levels of pro-inflammatory “adipokines”, especially leptin, resistin and visfatin, and less of the anti-inflammatory adiponectin [31,205,226]. Accordingly, a decreased adiponectin/leptin ratio is a marker of adipose tissue dysfunction [227]. Two large population-based studies in the US (n = 4009; 274) [228] and Spain (n = 11,540; 275) [229], respectively, showed that in apparently healthy adults, circulating leptin levels are positively related to age, BMI, female sex, fasting glucose, serum triglycerides, serum insulin, and CRP. In the Spanish subjects, those with obesity, hypertension, type 2 diabetes, hypercholesterolemia, and metabolic syndrome had higher leptin concentrations than their normal-weight counterparts [229]. This is in accordance with a myriad of studies dating back several years that demonstrate that insulin and leptin resistance are important features of obesity, type 2 diabetes, and low-grade inflammation [230]. Obviously, elevated leptinemia is a proxy for leptin resistance [231], which means that there is a (strong) leptin signal not being received or passed on, and therefore not being translated into an effect. Likewise, elevated insulinemia is a proxy for insulin resistance [232].

Interestingly, pregnancy is actually a physiological state of leptin resistance (increasing with gestational age) [233], and insulin resistance [234]. After delivery, this state of allostasis returns to homeostasis. By analogy, until further notice, there is no reason to assume that the immune system in obesity is irreversibly disrupted and therefore cannot become (partially) corrected by means of a healthy lifestyle. Accordingly, in the Spanish study mentioned above, leptin levels were lower in subjects who adhered to physical activity recommendations [229].

### 10.2. Leptin and Insulin Resistance as a Cause of Severe COVID-19

Overweight COVID-19 patients have elevated leptinemia, which correlates with pro-inflammatory cytokines, monocyte activation and severe disease [202]. The main risk factors for COVID-19 mortality, namely age, obesity, type 2 diabetes and impaired renal function [204], find a common denominator in leptin and insulin resistance. Accordingly, high plasma glucose—a consequence of insulin resistance—is a predictor of a poor COVID-19 prognosis. In a Chinese study of hospitalized COVID-19 patients, a fasting blood glucose ≥ 7.0 mmol/L at admission predicted an approximately 2.30-fold (hazard ratio) higher risk of death within 28 days. This prediction was independent of a pre-existing diabetes diagnosis [235]. In another study from China, with 941 subjects hospitalized for COVID-19, hyperglycemia was associated with ARDS, acute cardiac, renal and hepatic damage, as well as cerebrovascular accidents [236].

The poor COVID-19 prognosis seen at higher glucose levels is associated with the unregulated production of proinflammatory cytokines [237,238,239,240]. Interestingly, whereas glucose is pro-inflammatory, insulin actually has an anti-inflammatory effect, at least at physiological concentrations [238]. In vitro exposure of monocytes to a high glucose concentration causes numerous changes in gene expression, including the expression of pro-inflammatory cytokines and adhesion molecules [237]. Even before the COVID-19 pandemic, hyperglycemia during influenza A infection was known to be a possible trigger of a cytokine storm [239]. Elevated blood glucose levels cause a higher activity of interferon regulatory factor-5 (IRF5). IRFs are a family of master transcription factors that, upon infection, orchestrate the innate and adaptive immune systems by inducing the expression of various cytokines [241]. Hyperglycemia causes increased N-acetylglucosaminylation of IRF5 via the hexosamine biosynthetic pathway [242], which in turn can lead to the increased expression of several cytokines, possibly contributing to a cytokine storm [239]. The increased expression of IRF5 is also related to lymphopenia, and the specific loss of cytotoxic CD8+ lymphocytes and classical monocytes [241,243], as seen in patients with type 2 diabetes with severe COVID-19 [244]. IRF5 expression in classical monocytes is increased 2.1-fold in patients with and without diabetes admitted to the ICU due to COVID-19 [244]. Not only diabetes, but also aging, obesity and metaflammation, by leading to impaired glucose homeostasis, can potentially create the perfect storm for a SARS-CoV-2 infection [4].

Leptin maintains communication between the adipose tissue and the metabolic organs, such as the liver, muscles, pancreas and central nervous system, as well as the immune system [89]. As mentioned, leptin has a “providing” role in the use of energy in the immune system, and this role is impaired in obesity. The prevailing insulin and leptin resistance affects both the central nervous system and the periphery. Less well known is that leptin resistance also affects the immune system [88,89,200,205,245].

Under the circumstance of combined leptin and insulin resistance and increased plasma leptin and insulin levels, (part of the) immune system has also become insensitive to leptin and insulin. This has a negative influence on its functioning. Indeed, and as discussed before, insulin is a key regulator of T cell metabolism and T cell function. Without insulin signaling, T cells cannot be activated [177,182,183]. As for leptin, it is a key regulator of the innate and adaptive immune system through the modulation of immune cell metabolism, proliferation and activity [31,245]. The predominant role of leptin on the immune system is that of a pro-inflammatory cytokine. It activates pro-inflammatory cells, such as neutrophils, monocytes, NK cells, induces the differentiation of naive CD4+ T cells into Th1 cells, and indirectly promotes the production of pro-inflammatory cytokines, such as TNF-α, IL-2 and IL-6 [246]. However, the role of leptin is nuanced; it is necessary for early T-cell development and the late development of CD4+ T-cells, but not for CD8+ T-cells [247]. Occasionally, co-stimulators are also needed [246]. The influence of leptin on the various cells of the innate and adaptive immune system is now well documented [245]. Interestingly, in mice, obesity accelerates the involution of the thymus and constricts T cell diversity [248]. This influence may be a manifestation of leptin and insulin resistance in the immune system. The treatment of malnourished mice with exogenous leptin restored their immunosuppressive phenotype and thymic atrophy [245]. How in metaflammation the immune effects of leptin change under the influence of the chronic hyperleptinemia and leptin resistance is not yet known in detail. However, restoring leptin signaling in leptin resistance is not easy. The administration of leptin is effective in leptin deficiency, less effective when leptin is adequate, and downright ineffective in leptin abundance (i.e., leptin resistance) [177,233,245,249,250].

It is clear that insulin and leptin resistance cause imbalances in the immune response. In metaflammation, due to the important role of insulin and leptin in the activation of the immune system, the combination of resistance to its actions and their increased concentrations form the mechanistic core that leads to an immune system imbalance and severe COVID-19 following SARS-CoV-2 infection. It is appropriate to exercise nuance in the use of the word “resistance”. It suggests that all distinct functions of the hormone/cytokine in question are disrupted. However, that is not the case. One function may be disabled, but another may become over-stimulated due to the compensatory elevation of the hormone/cytokine. “Inhibition” or “stimulation” of functions can be different for the individual organs and even for the different cells in a single organ [230,251]. This is called “selective resistance”. This selectivity also leads to inter-individual differences with numerous individualized clinical phenotypes [230].

### 10.3. The Core of Severe COVID-19

In obesity, the insulin and leptin resistance occurring in immune cells can be traced to an increased SOCS1/3 [252]. SOCS3 expression in obesity is associated with decreased energy expenditure, increased food intake, increasing adiposity, and insulin and leptin resistance [253]. As previously mentioned, SOCS1/3 are the checkpoint regulators of the immune system that are “hijacked” by SARS-CoV-2. In obesity, SOCS1/3 are induced by the chronically increased signaling of pro-inflammatory cytokines, including leptin, via the JAK/STAT pathway. The elevated insulin levels also contribute. The elevated SOCS1/3 suppress both leptin and insulin signaling via negative feedback [252], and are therefore at the basis of insulin and leptin resistance [30,147,222,226,247,252,254,255,256,257,258,259]. Thus, the chronically moderately elevated circulating inflammatory cytokines, including leptin, originating from the inflamed adipose tissue compartment, cause SOCS1/3 to be induced in metaflammation. This deactivates the leptin receptor and also other SOCS1/3 sensitive cytokine receptors [147,247,257,259], and does not “authorize“ the use of energy for an immune response. The exact mechanisms by which obesity leads to decreased T cell function aren’t completely known. However, as mentioned, hyperleptinemia promotes chronic inflammation which ultimately contributes to T cell exhaustion; T cells from people with obesity have higher levels of exhaustion markers. The insulin receptor itself is also known to contribute to cell function [183], although whether T cells become insulin or leptin-resistant with long-standing obesity is still unclear. Since leptin signals through STAT3, hyperleptinemia likely contributes to heightened basal STAT3 phosphorylation in addition to an increased expression of SOCS3 and an exhaustion of T cell function, similar to the immune aging model described by Shen-Orr et al. [166], whereby elevated age-related inflammatory cytokines lead to a constitutive activation of STAT1 in T cells from older immunosenescent adults, which become unresponsive to ex vivo stimulation.

We now have the answer, in part, to the question why in people with obesity the T cells are not activated during an infection. Disabling insulin and leptin signaling does not grant permission to initiate an energy-expensive and highly damaging immune response. The state of metaflammation does not allow the consumption of energy in a part of the immune system. It forms the mechanistic explanation of the already mentioned observation that a “hot” inflammation (infection: permission) is catabolic, and a “cold” inflammation (metaflammation: no permission) is anabolic [260]. In the CNS, leptin resistance means that, despite high leptin levels and large fat reserves, the brain does not receive signals to stop food intake and to expend more energy, resulting in a further increase in obesity through a sense of hunger and reduced energy expenditure. This creates a paradoxical state of abundant energy reserves and a feeling of hunger. Mice that do not make leptin or do not have a leptin receptor for genetic reasons become obese, but at the same time display the immunological and endocrine deficiencies associated with starvation [246]. If a “hot” inflammation (read: infection) is superimposed on a chronic “cold” inflammation (read: metaflammation), part of the immune system is initially not allowed to expend energy for activation, the necessary “hot” reaction in case of infection starts slowly, an imbalance in the immune response arises, and the immune system will overreact in the second instance to make up for the delay. If the “hot” inflammation is caused by the very fast-striking SARS-CoV-2, then the accumulated delay and especially the subsequent overreaction can be deadly.

### 10.4. Reversing Leptin Resistance

In obese mice, an improvement of the slow immune activation was achieved through physical exertion. This was observed with influenza A infection. Physical exertion of infected non-obese counterparts resulted in an early reduction of the amount of virus and a modest inflammatory response. Physical exercise reduced the severity of infection in both obese and non-obese mice [261]. In healthy humans, regular physical activity is associated with better in vitro activation of immune cells in the peripheral blood following stimulation with microbial antigens [262], reduces inflammation [263], and improves insulin sensitivity [264]. Accordingly, a retrospective observational study conducted in Southern California, using a sample of 48,440 adults, has found that those who had been consistently physically inactive from March 2018 to March 2020 had a greater risk of hospitalization (OR 2.26; 95% CI 1.81 to 2.83), admission to the ICU (OR 1.73; 95% CI 1.18 to 2.55) and death (OR 2.49; 95% CI 1.33 to 4.67) due to COVID-19 than those who have consistently followed physical activity guidelines during that period [265]. Interestingly, a major acute exertion resulting in an energy expenditure of more than 800 kcal reduces leptinemia. Chronic physical exercise [266], and energy-restricted diets lasting more than 12 weeks [267], also significantly lower leptin levels, but this effect appears to be mainly related to the loss of adipose tissue [266]. As mentioned, there is a linear relationship between circulating leptin and the degree of adiposity [198,199,228,229]. The causal relationship between obesity, hyperleptinemia and leptin resistance was demonstrated in laboratory animals. Partial reduction of plasma leptin by neutralizing antibodies restored leptin sensitivity in the hypothalamus, decreased weight gain and increased insulin sensitivity [253].

Lowering SOCS3 is the rationale for experimental drug therapies to override leptin and insulin resistance. To this end, the effect of SOCS3 antagonists is being investigated [268]. However, due to the guarding role of the SOCS proteins in the inhibition of the immune response, such a treatment is not without risk. In the longer term, it is more logical and safer to remove the real cause of insulin and leptin resistance, which means preventing and reversing metaflammation through an improved lifestyle. This includes adequate sleep, stress management, the removal of various industrial toxicants and endocrine disruptors, smoking avoidance, regular exercise, and a healthy diet that contains specific nutrients (e.g., omega-3 fatty acids) and bioactive compounds (such as several phenolics) that inhibit the inflammatory response or induce its resolution, while also allowing for an appropriate anti-viral response (e.g., vitamins A, C, D, E, B6, B9 and B12, magnesium, iron, zinc, copper, and selenium) [6,39,42,218,269].

## 11. Comprehensive Summary and Discussion

Acute and chronic inflammation trigger different adaptive responses. Both classical acute inflammation and (chronic) metaflammation lead to insulin and leptin resistance and the activation and recruitment of immune cells. Classic inflammation, also called “hot” inflammation, and characterized by dolor (local pain), calor (local heat), tumor (local swelling), rubor (local redness) and functio laesa (disordered function), is associated with a higher consumption of energy (catabolism). Metaflammation, also known as “cold” inflammation, however, involves reduced energy expenditure (anabolism) [260]. There is something that opposes energy consumption. In the metaflammation of obesity, the inflamed adipose tissue is the driving force. The chronically slightly elevated proinflammatory cytokines (including leptin, which has been found to be elevated in COVID-19 patients [202,270,271,272]—Table 1) can cause SOCS1/3 induction [23,254], thus inhibiting the signaling of cytokines (such as interferons), leptin, and insulin [254,268]. “Resistance” arises, and that affects various organs, including parts of the immune system.

If a person with metaflammation becomes infected with SARS-CoV-2, initially no permission is granted to start an energy-consuming and damage-causing immune response. An early production of interferons types I and III is not forthcoming (Figure 1 and Figure 2). These have antiviral properties and thus prevent rapid virus spreading. By disrupting interferon signaling, SARS-CoV-2 adds a significant boost to this. The basal leptin and insulin resistance in the immune system that is prevalent in metaflammation qualify as a cause for the late immune response in obesity. This resistance arises because of the chronically elevated cytokines, including leptin, which induce SOCS1/3 and thereby suppress cytokine signaling. SOCS1/3 is also induced by the virus in a poorly understood manner. The delayed and weak antiviral response allows for a strong multiplication and spreading of SARS-CoV-2 (Figure 2). Major damage is caused by the virus and by the immune system that overreacts in the second instance. The result is a cytokine storm and a state of hypercoagulation, which are key factors in severe COVID-19. SOCS1/3 induction due to metaflammation is thus the prime suspect. However, SOCS1/3 are victims rather than perpetrators. They are critical to keeping the immune system in check. Indeed, knockout of SOCS1 in laboratory animals causes neonatal death from an unregulated inflammation and knockout of SOCS3 results in embryonic death [156]. In metaflammation, the induction of these SOCS proteins is caused mostly by an unhealthy lifestyle. The chronically elevated pro-inflammatory cytokines, leptin and insulin levels in obesity find a cause in an inflamed adipose tissue compartment. They drive the leptin and insulin resistance and the associated hyperleptinemia, hyperinsulinemia, hypertension, hyperglycemia, hyperlipidemia and other conditions that characterize metabolic syndrome. Lifestyle-induced metaflammation and associated diseases (such as type 2 diabetes, cardiovascular disease and certain cancers) have not been subject to evolutionary pressure. In Western societies, plasma leptin rises from adulthood [199,228,229]. However, this increase was not seen in a traditionally living population in Kitava (Trobriand Islands, Papua New Guinea). At all ages, the adults studied in the Kitava population [273] had lower plasma leptin levels than their peers in Sweden [273] and Spain [229]. This has been attributed mainly to their low BMI [273], but perhaps certain aspects of their non-Western diet likewise played a role [274]. Interestingly, Kitavans also had lower fasting serum insulin and CRP levels compared to a Swedish control population [275,276].

## 12. Conclusions

Superimposing an acute disturbance, such as a SARS-CoV-2 infection, on the allostatic state of metaflammation severely tests resilience. In the long term, metaflammation causes the “typically Western” conditions associated with metabolic syndrome. Severe COVID-19 and other serious infectious diseases can be added to the list of its short-term consequences. Therefore, preventive measures should include not only vaccination and the well-established actions intended to avoid infection, but also the appropriate dietary and lifestyle interventions aimed at improving body composition and preventing or even reversing metaflammation and leptin resistance.

## Figures and Tables

**Table 1:** Human studies finding higher leptin levels in COVID-19 patients.

Country, Author	Sample population	Conclusions
China		
Wang, J.; et al. [202]	* 12 healthy subjects (mean age: 48 ± 15.7 years; 50% males; BMI: 23.8 ± 2.9 kg/m^2^) * 21 mild COVID-19 hospitalized patients (mean age: 47.2 ± 15.7 years; 61.9% males; BMI: 23.69 ± 2.7 kg/m^2^) * 10 severe COVID-19 patients (mean age: 46.9 ± 16.5 years; 70% males; BMI: 25.3 ± 2.6 kg/m^2^)	Leptin was associated with greater monocyte activation, systemic inflammation, and disease progression in COVID-19 cases
The Netherlands		
van der Voort, P.H.J.; et al. [270]	* 31 COVID-19 patients in ICU requiring ventilation (mean BMI: 31 kg/m^2^ [range 24.8–48.4]) * 8 critically ill non-infected controls (mean BMI: 26 kg/m^2^ [range 22.4–33.5])	Higher serum leptin levels in patients with COVID-19, in comparison with controls
India		
Singh, R.; et al. [271]	* 10 healthy subjects (mean age: 48.14 ± 9.05 years; 74% males; BMI: 26.28 ± 2.52 kg/m^2^) * 15 individuals who recovered from mild COVID-19 (mean age: 38.07 ± 7.7 years; 100% males; BMI: 26.84 ± 2.63 kg/m^2^) * 34 moderate COVID-19 hospitalized patients (mean age: 48.21 ± 9.79 years; 75.86% males; BMI: 26.31 ± 2.29 kg/m^2^)	Increased leptin levels in moderate COVID-19 patients, compared to healthy subjects and individuals who recovered from mild COVID-19
Sweden		
Larsson, A.; et al. [272]	* 25 healthy subjects (median age: 57 years [range 47–68 years]; 76% males) * 222 severe COVID-19 hospitalized patients (median age: 64 years [range 24–86 years]; 79.28% males)	Leptin levels was higher in patients with COVID-19 at ICU admission, but it wasn’t associated with mortality

## Data Availability

Not applicable.
